# Transforming clinical data into wisdom

**DOI:** 10.1097/01.NUMA.0000719396.83518.d6

**Published:** 2020-11

**Authors:** Kenrick D. Cato, Kathleen McGrow, Sarah Collins Rossetti

**Affiliations:** Columbia University School of Nursing in New York, NY.; Microsoft Corporation in Redmond, Wash.; biomedical informatics and nursing at Columbia University in New York, NY.

Artificial intelligence (AI) is part computer science and part cognitive science, encompassing the phenomena of computers performing tasks that require human intelligence.^1^ Current interest in AI is motivated, in part, by recent developments in machine learning, in which algorithms learn from data without human direction.^2^ Machine learning is a group of different mathematical methods to build AI rules. Each of these methods has its strengths and weaknesses based on the types of data being analyzed. Some machine learning approaches, such as speech recognition when talking into the speaker of a smart phone or smart home device, are now mainstream.

Although we’ve been on a journey for decades to augment human capabilities, recently there’s been a dramatic rise in AI research in healthcare. AI has been brought to the forefront due to multiple factors, including the tremendous amounts of data that are captured and stored through electronic health records (EHRs), more availability of computer power, and significant innovations in AI tools and algorithms. The increase in AI availability and use in healthcare makes it imperative that both nurse leaders and nurses have a fundamental understanding of AI when used in conjunction with clinical models, critical-thinking skills, and evidence-based practice.

Graves and Corcoran identified the conceptual model that’s pivotal to nursing informatics known as the data, information, knowledge, wisdom (DIKW) framework.^3^ This framework has subsequently been used to define how nursing informatics impacts nursing practice and care delivery, and it aligns well with the American Nurses Association *Nursing Informatics: Scope and Standards of Practice,* which identifies nursing informaticists as assisting in the development of wisdom for all nurses.^4^ In addition to the use of knowledge, wisdom includes decision-making and applying nursing actions through the applicable use of data, information, and knowledge.^5,6^ In the healthcare setting, AI is often paired with a clinical decision support (CDS) application to move from knowledge to wisdom, which involves exercising judgment.^7^ The ability to use AI-assisted wisdom to solve problems and identify solutions is key for nurses to enable the delivery of nursing care that’s more precise and efficient, benefiting both nurses and patients.^8^

In this article, we explain the DIKW framework, review key concepts of AI and CDS, offer examples of real-world AI/CDS applications within the DIKW framework and how they can be used, and identify relevant questions to ask when investigating AI for use in clinical and operational scenarios.

## DIKW, AI, and CDS

The DIKW framework explains that data leads to information, information to knowledge, and knowledge to wisdom.^9^ This framework is particularly relevant when designing CDS tools to advance nursing practice to top of licensure for improved nurse decision-making. The DIKW framework can be used to categorize CDS tools, including those that are driven by AI. This framework is pyramidal, which implies that data are profuse and wisdom is rare. As defined in previous publications discussing this framework, data have little or no meaning in isolation, information is data plus meaning, knowledge is derived by discovering patterns and relationships between types of information, and wisdom is understanding and internalization of knowledge patterns and relationships. (See Table 1.)^10^ The DIKW framework isn’t just a way to understand how information can be processed; it’s also a means by which nurse leaders can strategically evaluate the relationship between AI and CDS.

AI generally refers to “machines that respond to stimulation consistent with traditional responses from humans, given the human capacity for contemplation, judgment, and intention...[these machines] make decisions which normally require human level of expertise.”^11^ West and Allen describe three characteristics of these systems, which “operate in an intentional, intelligent, and adaptive manner.”^12^ Intentionality is defined as algorithms designed to make decisions, typically using real-time data.^12^ Intelligent systems use underlying trends or patterns to inform decision-making.^12^ And an adaptive manner includes the ability to learn and improve decisions based on new information or pattern recognition.^12^ In the healthcare setting, AI is used with analytics to support CDS, improve operational efficiency, and for general cost effectiveness.^13^

CDS and AI aren’t one in the same. CDS provides clinicians with computer-generated clinical knowledge and patient-related information that’s intelligently filtered and presented at appropriate times to enhance patient care.^14^ In other words, AI can be thought of as a fuel to power more intelligent CDS. For example, imagine having a CDS alert for nurses to help them manage a patient with diabetes based on information from tens of thousands of patients and other nurses in a similar situation. Even though CDS interventions can be used to implement AI at the point of care, they don’t always leverage AI. In fact, a 2017 integrative review identified that only 25% of CDS targeting nurses uses real-time EHR data.^15^

An analysis of requests for development of CDS targeted at nurses identified that the majority of requests were information and knowledge related when plotted along the DIKW framework.^16^ There’s untapped potential for use of AI within CDS interventions that target risk assessment/risk reduction activities within the nursing process.^16^ These areas include AI-based CDS interventions that align with the wisdom concept from the DIKW framework and use, for example, predictive analytics, personalization of information presentation, patient preferences, and social and behavioral determinants of health to guide nurses’ understanding and internalization of knowledge patterns and relationships.^16^

Targeted CDS nursing interventions shouldn’t stop at the planning stage of the nursing process as many do currently.^16^ To impart wisdom--understanding and internalization of knowledge patterns and relationships--AI designed for nurses should be intentionally designed to use real-time data to recommend decisions, have intelligent recognition of patterns within nursing and patient care workflows and processes, and be adaptive by aligning with the cyclical nature of workflow activities in the nursing process.

**Figure 1:**
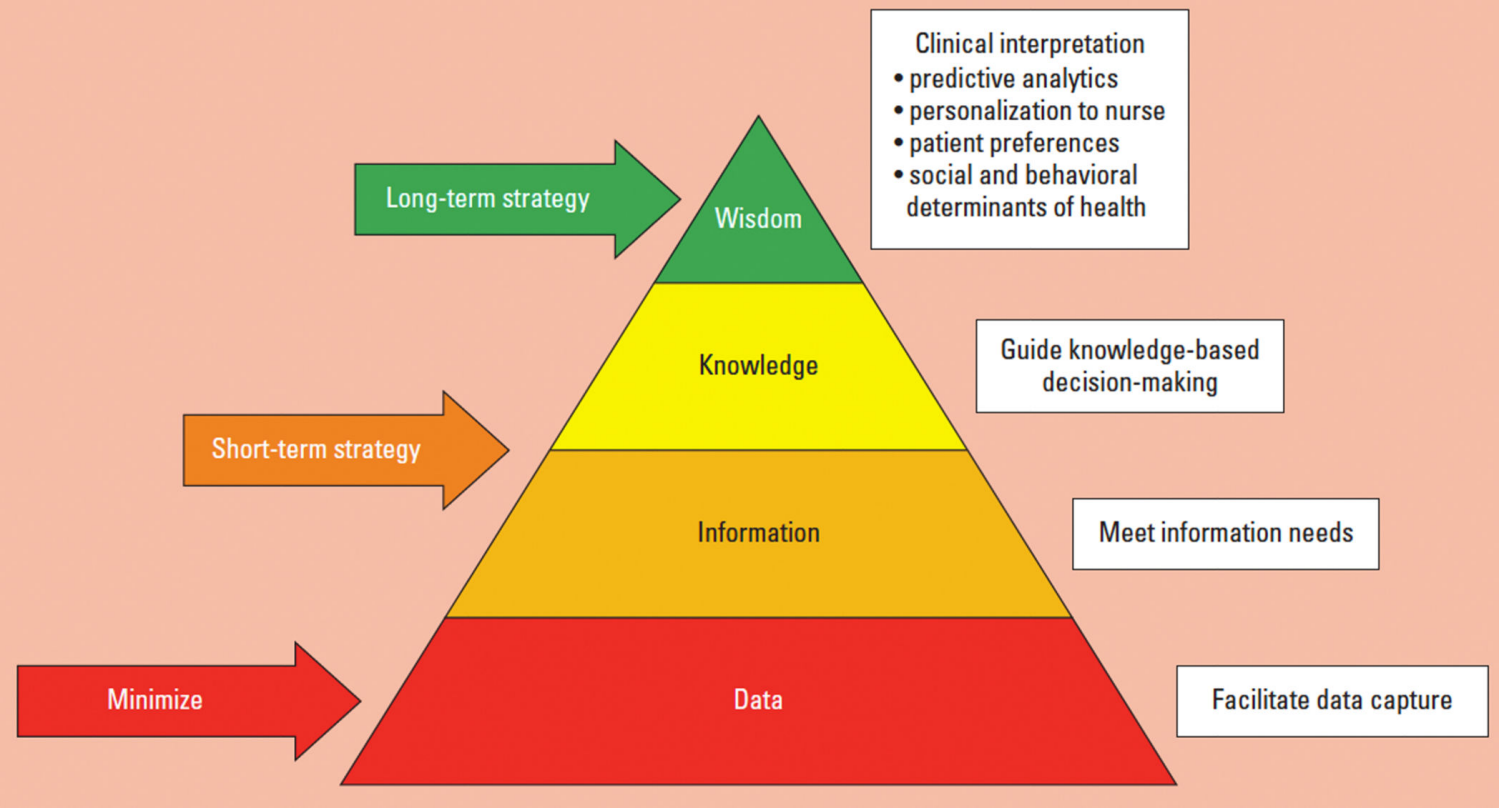
DIKW strategic plan

In healthcare, there’s been an increase in AI/CDS-based tools. Some of these offerings don’t solve critical nursing decision support needs. However, there are AI/CDS tools that nurse leaders can benefit from; for example, tools that provide predictive analytics for staffing and bed management, to name a few. The DIKW model can help leaders understand which of these AI/CDS tools will be useful in their organization. Figure 1 illustrates the hierarchical nature of AI/CDS applications in which there are data capture, data processing to meet information needs, clinical knowledge-based rules to guide decision-making, and exposed analytics in the form of wisdom to augment nurse decision-making.^16^

From a clinical user perspective, the bottom three levels of the application usually aren’t visible. The user often sees the top, fourth level in the form of an alert, dashboard, risk score, app, or other interface. However, when investigating AI/CDS applications, it’s essential to understand how all of the levels were developed and validated.

### Real-world examples

There are numerous examples of AI/CDS applications for nurses, such as the AI-based sepsis CDS alerts used in many EHRs today.^17–22^ There are also newer AI/CDS tools, such as the Communicating Narrative Concerns Entered by RNs (CONCERN) CDS system. CONCERN is an AI/CDS tool that predicts a patient’s risk of clinical deterioration, which is currently being used at Partners Health System, with a planned rolled out at NewYork-Presbyterian Hospital later this year.^23^ Both the sepsis CDS tool and the CONCERN CDS system illustrate how the DIKW approach can be used by nurse leaders to determine if these technologies make sense in their clinical setting.

A number of different types of AI rules have been built to trigger sepsis CDS alerts.^24^ The data that most of these sepsis alerts use are vital signs and lab results. Machine learning is then used to build AI-based rules to predict patients who are at risk for sepsis. The information generated is the meaning of different types of lab results and vital signs, and the knowledge is the predicted increased risk. The current sepsis CDS alerts don’t reach the wisdom level for two reasons. First, because the sepsis alerts are delivered by the EHR, there’s a false alarm or fatigue alert issue. This situation happens because no AI is perfect; many patients receive the sepsis alert, disrupting the nurse’s regular workflow when the patient clearly isn’t at risk for sepsis. Eventually, nurses get tired or fatigued by these alert-based disruptions and ignore the CDS altogether. Second, most sepsis alerts trigger 4 hours before it’s clinically clear that the patient is significantly deteriorating.^24^ This doesn’t allow clinicians much time to intervene and effectively treat the patient.

The CONCERN CDS identifies nursing documentation patterns that are a proxy of a nurse’s concern for patient deterioration and generates a predictive early warning score. The CONCERN CDS consists of two main components: the decision engine, which uses AI models to analyze the content and patterns of nursing EHR data (such as flowsheet entries and nursing notes), and front-end interfaces that display the predicted CONCERN levels (green, yellow, red), with red indicating that a patient is at the highest risk for deterioration. A web application provides tracking of the CONCERN level over time and facilitates transparency of the AI models by displaying what factors contributed most to the current calculation.

The result of more than 8 years of qualitative and quantitative research involving clinician input at every stage, the CONCERN CDS is built with the DIKW framework in mind. Data capture is automatic from the EHR, with the AI model using data entered during the normal nursing workflow. The nursing data used include patterns and content of nursing notes, medications administration record information, and flowsheet entries. The system continuously monitors data inputs from patterns in nursing documentation and flowsheets to model performance. Alerting tools let the programmers who maintain the system know when expected data feeds are missing or the model output is different than expected.

**Figure 2:**
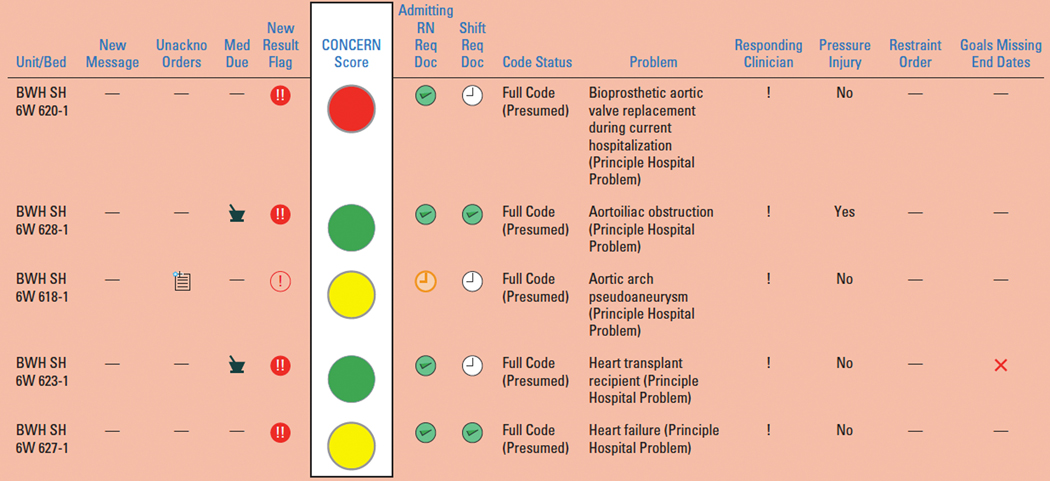
Sample patient list

Information generation is based on focus group and simulation testing with nurses and physicians. An example of knowledge representation is the green, yellow, red scoring system that indicates the patient’s risk of deterioration. When the CONCERN CDS was being developed, nurses and physicians indicated in focus group and simulation testing that colored levels for risk of deterioration would be the most clinically meaningful. Also, the setting-specific (acute care versus ICU) CONCERN AI models are optimized to identify patients who score in the yellow category. Clinicians reported that patients at the lowest and highest risk for deterioration are much easier to identify. Patients at the yellow level are at increased risk for clinical deterioration but haven’t started showing signs yet. Figure 2 shows a sample patient list in which users can quickly see a patient’s CONCERN level in their workflow.

**Figure 3:**
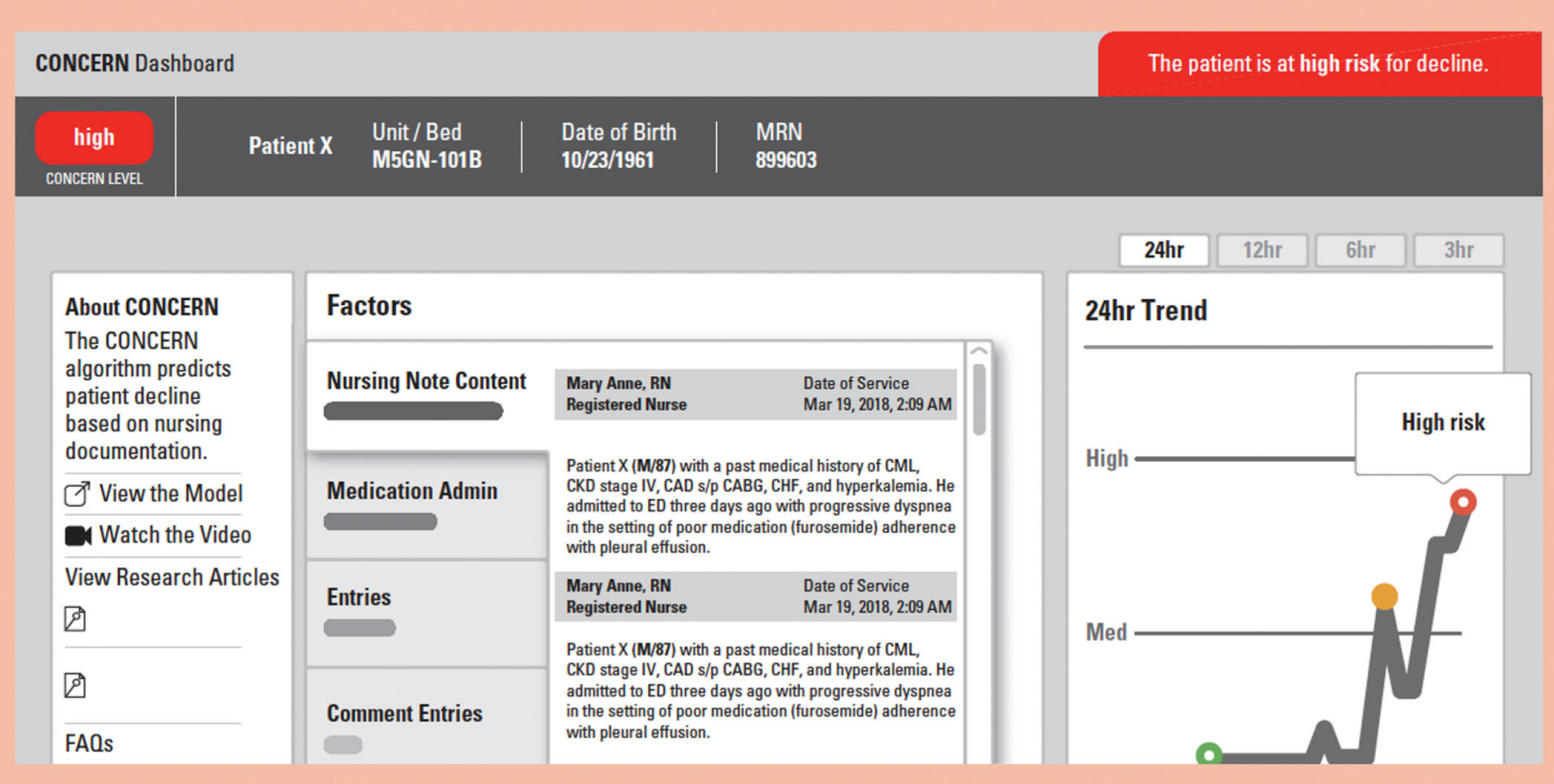
CONCERN web application

The CONCERN CDS is designed to augment clinical expertise instead of replacing it. Figure 3 shows the main CONCERN web application, which opens when a clinician clicks on a CONCERN level in the patient list. The main decision support part of the CONCERN CDS system is focused on visualizing trends in patient risk. The factors portion of the CONCERN CDS provides AI transparency, explaining what elements of the patient record contributed to the risk calculation. Additionally, a major goal of CONCERN CDS system is to support interdisciplinary communication by providing the relevant information (notes, medications administered, flowsheet entries) for patients at increased risk for deterioration in a centralized, accessible location many hours before the patient crashes. The CONCERN score has been shown to trigger up to 24 hours before a clear deterioration event.

### Questions for nurse leaders to ask

With the abundance of data inside and outside of the EHR, AI/CDS applications are being used more frequently by nurses to provide care for patients.^25^ However, research indicates that the success of new AI/CDS systems requires careful planning, evaluation, and decision-making.^15,26–28^ AI/CDS applications are often presented as solutions to pressing clinical problems. The DIKW framework can help nurse leaders organize strategic planning and prioritization of potential AI/CDS applications, helping connect clinical practice with informatics solutions and revealing potential nursing practice or informatics gaps for proposed CDS.^16^ Table 2 describes a number of questions based on the DIKW levels that nurse leaders should ask about AI/CDS systems.

### Strategic implementation

AI-powered CDS tools offer great promise in helping nurses to provide higher quality, safer, and more efficient care. It’s imperative that nurse leaders understand the key concepts of AI and CDS. The DIKW framework and relevant questions support nurse leaders in thinking strategically about current and future implementation of AI/CDS applications.

## Figures and Tables

**Table 1.** Data-Information-Knowledge-Wisdom Conceptual Framework (DIKW) Definitions^5,11^

Concept	Definition
Wisdom	Understanding and internalization
Knowledge	Derived by discovering patterns and relationships between types of information
Information	Data plus meaning
Data	Little or no meaning in isolation

**Table 2.** Questions that a Nurse leader should ask about potential AI/CDS tools

DIKW level	Question	Explanation
**Data**	What data is used in the AI/CDS tool?	It is important to understand the required datasources. Also, what types of transformations that are done on the data that might change it’s clinical meaning. For example, what are the cutoffs for how results are categorized?
	How is it captured? Does this data capture fit into the existing clinical workflow?	It is important to know if data capture will add any additional burden to clinicians. Also, will clinicians workflow be impacted by required data capture?
	Is there an appropriate life cycle plan for the CDS?	Data capture is dynamic, with continuous changes in the configuration. For example, forms in the EHR will be updated, created, or retired. Is there a similar life cycle plan for the CDS.^18^ How will the CDS handle EHR changes? Is there de-implementation planning?
**Information**	Does the AI/CDS information take into account the clinical context?	The CDS should be agile enough to adapt to changes in clinical settings. For example, the same models should not be applied across settings where workflow and policies are different, for example acute care versus intensive care settings.
	Does the information produced make clinical sense and have clinical relevance?	You should ensure that clinicians review the decision support and validate the clinical appropriateness and relevance of the CDS recommendations.
**Knowledge**	Does the AI/CDS help to solve a clinical problem. What were the examples that were used to teach the model?	For, AI/CDS, the products of the model are only as good as the data and information that helped create it. ^22^
	Does the clinical decision support fit nursing processes?	Effective CDS should adhere to nursing science and processes.
**Wisdom**	Is the AI/CDS augmenting or taking over decision making?	The goal of effective AI/CDS should be to aid in decision making not replace the clinician particiapation.^23^
	Is the AI explainable to the clinician?	AI/CDS will not be effective if clinicians don’t understand how the recommendation are produced.^24^
	Is the required short-term and long-term training in place?	Continuous and effective training is required to be confident that AI/CDS users know what recommendations represent and how to use them in their clinical practice?^25–27^

## The CONCERN AI/CDS: An Example

There are a number of exciting examples of these new AI/CDS applications, among them is the Communicating Narrative Concerns Entered by RNs clinical decision support CONCERN CDS system. The CONCERN CDS is an AI/CDS that uses technology that has identified nursing documentation patterns that are a proxy of a nurses concern for deterioration and includes these proxies as features in a predictive early warning score. The CONCERN CDS is consists of two main components. First, the decision engine that uses AI models to analyze the content and patterns of nursing EHR data (e.g. flowsheet entries and nursing notes ). Second, front-end interfaces display the predicted CONCERN levels (green, yellow, red), with red indicating a patient is at highest risk for deterioration. A web application provides tracking of the CONERN level over time and facilitates transparency of the AI models by displaying which factors contributed most to the current calculation.
